# Draft Genome Sequence of the Butanoic Acid-Producing Bacterium Clostridium luticellarii DSM 29923, Used for Strong Aromatic Chinese Liquor Production

**DOI:** 10.1128/genomeA.00377-18

**Published:** 2018-05-03

**Authors:** Anja Poehlein, Rica Bremekamp, Veronika Theresa Lutz, Lisa Maria Schulz, Rolf Daniel

**Affiliations:** a Genomic and Applied Microbiology and Göttingen Genomics Laboratory, Institute of Microbiology and Genetics, Georg-August University of Göttingen, Göttingen, Germany; b Members of the Applied Bioinformatics in Microbiology Course of the Microbiology and Biochemistry MSc/PhD Program, Georg-August University of Göttingen, Göttingen, Germany

## Abstract

The strictly anaerobic, Gram-positive bacterium Clostridium luticellarii, which has straight or slightly curved rod-shaped cells, polar endospores, and peritrichous flagella, is used for the production of strong aromatic Chinese liquors. C. luticellarii is able to produce butanoic acid. The draft genome sequence consists of 3.757 Mbp, including 3,632 predicted protein-encoding genes.

## GENOME ANNOUNCEMENT

Chinese liquor production is a unique fermentation process, and butanoic acid is an important component for this aromatic beverage (1). Butanoic acid is produced by Clostridium luticellarii strain DSM 29923, which was first characterized in 2015 (1). The strain uses palatinose, l-fructose, β-hydroxybutyric acid, l-rhamnose, and α-ketobutyric acid as main carbon sources. The organism was originally isolated in the province of Sichuan, China, during batch brewing in mud cellars, which allows the production of strong and aromatic liquors (2). To gain deeper insights into the role of microorganisms in industrial production of Chinese liquors, the genome of C. luticellarii DSM 29923 was sequenced and analyzed.

Chromosomal DNA of C. luticellarii DSM 29923 was isolated using the MasterPure Complete DNA purification kit, according to the instructions of the supplier (Epicenter, Madison, WI). Illumina paired-end sequencing libraries were generated from the isolated DNA, as recommended by the manufacturer (Illumina, San Diego, CA). Sequencing was performed by employing the MiSeq reagent kit version 3 and the MiSeq sequencing platform, as described by the manufacturer (Illumina). Quality improvement of the recovered reads was obtained with Trimmomatic version 0.36 (3). The recovered high-quality reads (2,590,302 paired-end reads) were assembled by applying SPAdes genome assembler software version 3.11.1 (4). Assembly resulted in 165 contigs (>500 bp), with an average coverage of 134-fold. Qualimap version 2.2.1. was used to validate the assembly. The genome (3.757 Mbp) had a GC content of 30.96%. The software tool Prokka (5) was employed for automatic gene prediction, which yielded 3,632 predicted protein-encoding genes, of which 917 encoded hypothetical proteins. In addition, 68 tRNAs, 8 rRNAs, and 1 transfer-messenger RNA (tmRNA) were predicted. The genome contains 29 putative genes coding for antibiotic resistance, including 25 multidrug resistance genes. Furthermore, 18 putative protein-encoding genes linked to clustered regularly interspaced short palindromic repeat (CRISPR)/Cas systems were identified. Genome analysis also revealed the presence of the *asc* gene cluster, which is characteristic for the Wood-Ljungdahl pathway (6). Interestingly, organisms containing this conserved cluster are able to grow on H_2_ and CO_2_ (6); however, this feature has not yet been described for C. luticellarii DSM 29923. Potential genes encoding a butyrate kinase and phosphate-butyryl transferase, which are typically involved in butanoic acid production, were not found. However, the presence of genes encoding butyrate-acetoacetate coenzyme A (CoA)-transferase subunits A and B in the genome indicated production of butanoic acid in C. luticellarii DSM 29923 via this enzyme.

### Accession number(s).

The whole-genome shotgun project has been deposited at DDBJ/EMBL/GenBank under the accession number PVXP00000000. The version described here is version PVXP01000000.

## References

[1] Wang Q, Wang C, Li C, Li J, Chen Q, Li Y 2015 *Clostridium luticellarii* sp. nov., isolated from a mud cellar used for producing strong aromatic liquors. Int J Syst Evol Microbiol 65:4730–4733. doi:10.1099/ijsem.0.000641. 26420591

[2] Wang CD, Chen Q, Wang Q, Li C, Leng Y, Li S, Zhou X, Han W, Li J, Zhang X, Li Y 2014 Long-term batch brewing accumulates adaptive microbes, which comprehensively produce more flavorful Chinese liquors. Food Res Int 62:894–901. doi:10.1016/j.foodres.2014.05.017.

[3] Bolger AM, Lohse M, Usadel B 2014 Trimmomatic: a flexible trimmer for Illumina sequence data. Bioinformatics 30:2114–2120. doi:10.1093/bioinformatics/btu170. 24695404 PMC4103590

[4] Bankevich A, Nurk S, Antipov D, Gurevich AA, Dvorkin M, Kulikov AS, Lesin VM, Nikolenko SI, Pham S, Prjibelski AD, Pyshkin AV, Sirotkin AV, Vyahhi N, Tesler G, Alekseyev MA, Pevzner PA 2012 SPAdes: a new genome assembly algorithm and its application to single-cell sequencing. J Comput Biol 19:455–477. doi:10.1089/cmb.2012.0021. 22506599 PMC3342519

[5] Seemann T 2014 Prokka: rapid prokaryotic genome annotation. Bioinformatics 30:2068–2069. doi:10.1093/bioinformatics/btu153. 24642063

[6] Poehlein A, Cebulla M, Ilg MM, Bengelsdorf FR, Schiel-Bengelsdorf B, Whited G, Andreesen JR, Gottschalk G, Daniel R, Dürre P 2015 The complete genome sequence of *Clostridium aceticum*: a missing link between Rnf- and cytochrome-containing autotrophic acetogens. mBio 6:e01168-15. doi:10.1128/mBio.01168-15. 26350967 PMC4600107

